# The Hospital. Nursing Section

**Published:** 1904-06-04

**Authors:** 


### The Hospital.

**Nursing Section.**

Contributions for this Section of "The Hospital" should be addressed to the Editor, "The Hospital" Nursing Section, 28 & 29 Southampton Street, Strand, London, W.C.

No. 923.—Vol. XXXVI. *SATURDAY, JUNE 4, 1904.*

### Notes on News from the Nursing World.

### THE PAY OF ARMY NURSES.

Rules for applying the scale of pay authorised for members of Queen Alexandra's Imperial Military Nursing Service by Paragraph 2 of Army Order 87 this year have been approved by the Army Council. The first is to the effect that nurses who are already in receipt of the minimum, or more, of their class in the new scale are to continue in receipt of such rate of pay, and may receive increments under the new scale from the next anniversary of their old increment date. The second decrees that nurses who were in receipt of less than the minimum rate of their class in the new scale shall come under that scale from the date of the Royal Warrant, April 19, 1904, receive increments thereafter on the anniversary of that date.

### MISS BALFOUR AND DISTRICT NURSING.

At a meeting held in the County Buildings, Haddington, the Master of Polwarth in the chair, a scheme having in view the amalgamation and extension of existing nursing associations in the county, was discussed. The chairman having pointed out the advantages of constituting a nursing association for the whole county, and announced that several subscriptions had been received, Miss Balfour, sister of the Prime Minister, and president of the association in the eastern division of the county, strongly commended the scheme. It was ultimately unanimously agreed to adopt it, and on the motion of the chairman, Miss Balfour was appointed president. The new organisation is fortunate in making a start under such favourable conditions.

### ROYAL BRITISH NURSES' ASSOCIATION.

The annual meeting of the Royal British Nurses' Association will take place at the Imperial Institute, South Kensington, by permission of the authorities, on Monday next, June 6th, at 4.30 p.m. Princess Christian has graciously intimated her intention to preside. As the meeting occurs so soon after the conversazione there will be no social gathering of members afterwards.

### GUY'S HOSPITAL LADIES' ASSOCIATION.

The ninth annual general meeting of Guy's Hospital Ladies' Association was held in the Court-room, Guy's Hospital, on Saturday last. The President of the Association, Princess Christian, presided, and was received by Mrs. Benyon and Mrs. Cosmo Bonsor, and amongst those present were the Countess of Bective, Lady Edward Spencer Churchill, Lady Arthur Hill, the Hon. Mrs. Talbot, Lady Perry, Lady Fripp, Mrs. Frederick Taylor, Mrs. Galabin, Mrs. Hale White, Mrs. Symonds and Miss Swift, matron of the hospital. In the annual report which was read by the honorary secretary, attention was drawn to the increased success of the Association during the past year under the presidency of Princess Christian. It contained a recommendation from the Committee that £150 should be voted from the funds to support three beds in the maternity ward. It was also announced that two new branches had been instituted during the year, and that the number of garments made by members of the Association for the use of patients had considerably increased. The reports of the fourteen local branches were read and proved that all were in a highly satisfactory state. The Countess of Bective and Mrs. Arbuthnot Lane were elected to fill the vacancies on the Committee caused by the retirement of Lady Gull and Miss Swift.

### COLONIAL NURSING ASSOCIATION.

At the annual meeting of the Colonial Nursing Association next Wednesday the Duke of Marlborough will represent the Colonial Department, the Colonial Secretary being unable to attend. The other speakers, besides Earl Grey in the chair, will be Lady Lugard, Lord Westmeath, Sir Charles Bruce, and Sir Colin Scott-Moncrieff. Mrs. Debenham has been succeeded as secretary of the Association by Miss M. C. Dalrymple-Hay.

### MALE NURSES AND GENERAL HOSPITALS.

A certificated male nurse gives in another column a reason for the attitude of indifference which is observed by male nurses in reference to State registration. Indifference is by no means confined to one sex, but our correspondent's reason is extremely cogent. He is satisfied that if registration were adopted, the standard of training of many men who are engaged in nursing would be found sadly wanting, and they would be compelled to substitute for the word "nurse" that of "attendant." In order to remedy this condition of affairs, he proposes that the great general hospitals should admit male probationers into their training schools, and he avers that it would soon be found that they are of incalculable value. He makes light of the practical difficulties which, it has been contended, would arise if both sexes worked together in the same ward. But there is an obstacle in the way of any considerable multiplication of male nurses which we do not think he recognises. Nursing as an avocation does not offer the same inducements to young men of education as it does to young women of the same class, and we doubt if it ever will do so.

### NURSING AND THE IRISH LANGUAGE.

At the annual meeting of the Children's Hospital, Harcourt Street, Dublin, Colonel Dopping, who moved the adoption of the report, created considerable amusement by expressing the opinion that the people of the county would "be very much better employed in learning nursing than in learning Irish." No doubt the comparison was suggested to Colonel Dopping by the fact that the Dublin Corporation and County and District Councils have passed a resolution that not a single individual shall pass an examination unless he knows Irish. While sharing Colonel Dopping's enthusiasm as to the importance of nursing, we do not imagine that there is room for the wholesale accession to the number of nurses which he seems to contemplate.

### THE LAW OF KINDNESS.

With the full particulars of the case of an asylum attendant who has been fined at the South Western Police Court for smacking the face of a patient at the Middlesex County Asylum before us, we are disposed to think that the authorities might have been content to dismiss her. It was stated by the solicitor who represented the visiting committee of the asylum that the smack did not produce any great physical discomfort, and merely brought the colour to the cheek of the patient; the magistrate before whom the charge was heard said that the assault was of a slight character; and it appears that during the fifteen months the defendant had been at the asylum as probationer and nurse, no other complaint had been made against her. Undoubtedly her dismissal was absolutely essential, whether in the interests of discipline or of the patients. We are aware that it was pleaded on behalf of the offender that she lost her temper momentarily because the patient caught hold of her shawl and endeavoured to tear it; but there is great force in the plea that people who lose their temper, even momentarily, are not proper persons to have charge of lunatics. We are glad to learn that the law of kindness is the fundamental law on which the Middlesex Asylum has been conducted ever since it was established. This is as it should be, and if the twofold punishment of a transgressor of that law tends to emphasise the necessity for obedience to it, so much the better.

### SALE OF WORK AT NORTHAMPTON.

For the third year a sale of work on behalf of the District Nursing Association at Northampton, organised by the Queen's Nurses' Needlework Guild, has been held. For some weeks previously the nurses, district and private alike, had worked enthusiastically in leisure hours in order to promote its success. Considerable help was given by several Northampton ladies, including four of the Institute's former nurses now retired from the profession. Amongst the earliest arrivals were Mrs. Cooper, wife of the High Sheriff of Northamptonshire and chairman of the Nurses' Home, Lord Erskine, Sir Frederick and Miss Gunning, and several other members of the committee. A ladies' string band played at intervals, and a concert was also given in the nurses' sitting-room. The dining-room was set out as a tea room. £35 was realised by the sale. In addition to this the Guild had collected over £20 in subscriptions and donations, and they hope soon to have their ambition gratified. Their ambition is to have the Queen's Nurses' staff increased to seven. At present there are but five, and these are quite insufficient, however they may exert themselves, to meet the wants of the sick poor in the town.

### THE THREE GRADES IN WILTSHIRE.

At a public meeting of the Wiltshire Nursing Association held at Salisbury the other day Dr. Ord said that the passing of the Midwives' Registration Act had made the question of providing midwives very pressing. He pointed out that the women who practise midwifery in villages are practically all untrained. Why they had taken up their present mode of living one could hardly say, as in many cases their unfitness for the work was obvious. They had no theoretical knowledge; and their practical knowledge was largely gained at the expense of mother and child. In his opinion the only wonder was that there were so few fatalities. There were, however, he admitted, among these women a certain number with a natural proclivity for nursing, and it was exactly these that the Wiltshire Nursing Association would be able to help. The object of the organisation itself, Lady Radnor subsequently informed the meeting, was to encourage three grades of nurses, viz, the highly-trained, fully-certificated nurse for towns; village district nurses with not less than six months' general and maternity training; and cottage resident nurses with not less than six months' general and monthly training and midwifery certificate if desired. It is intended that the women with "a natural proclivity for nursing" shall belong to the third grade.

### THE SUPERANNUATION ACT AND THE AGE LIMIT.

At the spring meeting of the Gloucester and Somerset Poor Law Officers' Association, a letter was read from the nurses in the mental wards of the Eastville Workhouse, Bristol. The writers, in view of the possible amendment of the Superannuation Act, suggested that the age limit should be lowered. At present a nurse can only retire and claim superannuation at the age of 60, and it is urged that in consequence very few nurses join the Superannuation Fund. It was proposed that the age limit should be reduced to 50 or 55, and the proposal has been remitted to the National Poor-law Officers' Association for consideration. We can quite understand that the Nurses at the Eastville Workhouse who spend from 14 to 16 hours in the wards daily, do not feel that they can continue to do this until they are 60. But a demand from nurses themselves that they should be retired and superannuated at 50 should not be lightly put forward, because it is sure to encourage the preposterous idea that a nurse above that age is never equal to her work.

### THE TYRANNY OF THE GRANDMOTHER.

In the first number of the *Queen's Nurses' Magazine*—which is to be published three times a year, and judging by its contents deserves success—Miss Amy Hughes enlarges upon "Some Responsibilities of Queen's Nurses." Miss Hughes contends that every Queen's nurse has her part, however small, to play in dealing with such problems as the housing of the poor, improved sanitation, and the right feeding of infants and young children. She admits that the task is difficult, and that whether the nurse is working in town or country, she is face to face with the ignorance and prejudices of generations. Miss Hughes says: "We hear much of the despotic rule of mothers-in-law in Eastern households, but it is equalled in many a British home by the tyranny of custom exercised by the grandmother of the family." She maintains, however, that even the tyrannical grandmother can be conquered, and that a Queen's nurse who has realised the duties of her citizenship in common with her patients, will not be satisfied until she has discharged them.

### THE SALARY OF A SUPERINTENDENT NURSE.

The Colchester Guardians have decided to raise the salary of the superintendent nurse of the Workhouse Infirmary immediately from £40 to £45, and to add another £5 in twelve months' time. Before this determination was arrived at, however, a considerable discussion took place on the report of the Infirmary Committee who recommended the increase. One of the Guardians objected to the proposal on the ground that the superintendent nurse had not asked for an increase of salary, and said that it would be quite time to consider the matter when she made an application. Exception was taken to this view by two lady Guardians, one of whom affirmed that the Board knew that the superintendent nurse had been trying to better her position, and both expressed their conviction that it would be economy to retain the services of Miss Bradbury. In fact, it was generally maintained that under the auspices of Miss Bradbury, who was described as a particularly good officer, and a capable superintendent, the nurses are trained so well that if they had a medical examination they would be able to pass. We think that in these circumstances it was alike politic and gracious on the part of the Guardians to increase her salary spontaneously.

### CONTINUED SUCCESS AT DARLASTON.

The third annual report of the Darlaston Nursing Institute, which was adopted at the meeting of subscribers, shows that it continues to meet with satisfactory support. The health of the town during the last twelve months having been generally good, the two nurses were not fully employed, but they nevertheless paid upwards of 6,000 visits. The income for the year was £342 as compared with £340 in the previous year, and the expenditure £205 against £199. Working men's contributions still further increased to the extent of nearly £16, and most of the works in the town now subscribe to the association, which is in the happy position of possessing a balance in hand of £381, of which £200 is placed on deposit account at the bank. At the close of the meeting a discussion took place with regard to a proposal that the nurses should be allowed to attend private cases in their spare time, but it was very wisely decided to let the matter alone, at any rate for another year. The society has done so well thus far that it would be a great pity to make a departure which is always risky, and often ends in disaster.

### SOCIAL GATHERING FOR BRIGHTON NURSES.

The Dowager Countess of Tankerville has invited the nurses of Brighton and Hove, and any private nurses from London and the provinces who may be in Brighton on duty or pleasure next week, to attend a social gathering on Friday, June 10, in the Royal Pavilion, Brighton, from 2.30 P.M. to 4.30. The programme will embrace music, addresses by Dr. Torrey of Chicago, and afternoon tea.

### THE NURSES OF PORTSMOUTH PARISH INFIRMARY.

The number of nurses of the Portsmouth Parish Infirmary who were examined by Dr. Bryant, of Guy's Hospital, this year was 34. Sixteen third-year nurses presented themselves, and all obtained a sufficient number of marks to pass the examination. Miss Noad obtained over 75 per cent. of the possible number of marks, and Dr. Bryant affirms that all of these nurses showed a good practical knowledge of their work. Eight second-year nurses were examined and secured a sufficient number of marks to pass; and eight out of ten among the first-year nurses were successful, Miss Rosengrave obtaining over 75 per cent. of the possible number of marks.

### TWO MEDICAL MISSIONS IN BERMONDSEY.

It appears from a communication forwarded to us by the Secretary of the Medical Committee of the Church Missionary Society that the Bermondsey Medical Mission for Women and Children, to which we referred last week, has not taken the place of the Church Missionary Medical Training Mission, but is an additional movement, started by the late medical superintendent of the latter, which has been going on for three years. We are afraid that the existence of the two organisations with somewhat similar titles will be rather confusing, but if so the original institution cannot be blamed. Still its medical officer prescribes for the sick poor on the same days and at the same hours as heretofore, still she visits the sick at their own homes, and the practical work of surgical dressing under the superintendence of the doctor and nurse, is still a feature of the Church Missionary Training Home and Medical Mission.

### A SUCCESSFUL BAZAAR AT HANLEY.

The Hanley Nursing Society benefit to the extent of no less than £144 by the sale of work which has just been held in the town at the rooms of the Young Men's Christian Association. The money has come at a time when it was very urgently needed. Last year's report shows that the outlay was £300 as against an income of £168. The committee have also, as a matter of necessity, decided to engage a third nurse, which will involve an expenditure of quite another £100 a year. But the President of the Society is confident that the additional amount can be raised. It should, however, be raised in subscriptions, which will thus require to be more than doubled during the current year.

### SHORT ITEMS.

In consequence of difference of opinion on the part of the committee of the Sutherland Nursing Association, the Duchess of Sutherland has retired from the office of president.—At the recent examination of the London Obstetrical Society K. Marson and L. Till were successful. During the past year nine candidates, trained at Stoke Union Hospital, have gained the L.O.S. certificate.—Miss A. A. Tinker, district nurse of Hyde and Davenham, Cheshire, was married last month to Mr. Felix Thomas, of the Nursing Institute, 173 High Street, South Manchester.

### The Nursing Outlook.

"From magnanimity, all fear above;
From nobler recompense, above applause,
Which owes to man's short outlook all its charm."

### THE AMERICAN NURSE.

We are just welcoming to our shores a party of some forty American nurses who are on their way to Berlin to attend the International Congress of Women, particularly as regards the section on nursing, and under these circumstances it is interesting to glance at the position held by the nurse in the United States.

When an American nurse is off duty there are no means of telling what her profession may be: she wears no outdoor uniform, she is not a woman apart, or a woman of one idea, she is no more likely to discuss hospital questions than any other interesting subject. Except in the case of nurses who belong to some Church organisation, and are more like deaconesses the American nurse accepts her work as a profession rather than as a vocation, and she is exceedingly business-like in all her arrangements. The conditions of training are much the same as they are in England—that is to say, very varied. At the Johns Hopkins Hospital, for instance, the probationers are paid no salary, but they only work eight hours a day, and receive excellent educational instruction; at the other end of the pole there is a "nursing college" which professes to instruct the public by correspondence in a few months for a fee, and then issues a diploma. On the whole the three years' training with small pay, a ten-hours day and rather haphazard instruction prevail. But there is an interchange of probationers between the maternity hospitals of New York and the Children's Hospital of Boston, which shows how it might be possible for smaller hospitals in England to form training schools by the "group" method.

The American nurse after she has "graduated" is a very independent person, taking her own cases and her own fees, but generally very loyal to her training school. It is usual to have alumnæ associations in connection with the schools, and through these the graduate nurse keeps in touch with her comrades and with her work. Miss Mary Thornton, who is in charge of the party at present in England, is secretary to the "Nurses' Associated Alumnæ of the United States." The private nurse is generally supplied through a "Registry," which is merely a mutual convenience for doctors and nurses, and which is in no way an institution with officials who control the nurses. For instance, at Albany the "Van Ransselaer Building" was erected by a doctor especially for nurses, and arranged to suit them. But each nurse is merely a tenant occupying a set of rooms and is entirely independent. The building has a telephone and a porter and a confectioner's shop on the ground floor where meals can be had; yet it is not an institution, and is most certainly not a charity. If there is anything which can mar the charming politeness of the American nurse, it is to connect any of her work with that word charity; and she rather looks down on the English nurse because she is not as proud and independent as herself.

The question of social position does not arise in a country where the only schools are "public" schools and private schools are unknown. Where all have been children together, receiving a free education in the common schools, it matters little if when their paths part one becomes a nurse, another a teacher, and another a "lady" who lives on the labours of others, without contributing her quota to the world's work. The nurse in America stands on the same honoured plane as all other women—but she is held in no special popularity, has no extra privileges. Probably she would say that she does not want these things, but still she listens earnestly to the tale of how Queen Victoria received the Jubilee nurses, and Queen Alexandra the Pension Fund nurses. It is, perhaps, scarcely wonderful that in her democratic zeal she should make the mistake of not appreciating the thorough work on behalf of the best interests of nurses here which has been done by Queen Alexandra. It is easy to mislead those who are not conversant with the conventions of the country they visit for some few days only.

The American nurse is extremely hospitable, and no English nurse who has ever crossed the Atlantic but has been struck with the welcome she has received. Therefore we are glad to learn that on this occasion the party of nurses from the United States are to receive hospitalities in England. A committee, consisting of Mrs. Bedford Fenwick, Miss Isla Stewart, and a few other ladies, will devote Monday next to their entertainment, and propose to engage two brakes to drive the nurses round London to show them various places of interest; and in the evening a dinner organised by the same ladies will be held at the Criterion Restaurant, Miss Stewart in the chair. Miss Debenham, of St. Andrew's House, has also invited the visitors and the committee to luncheon at two o'clock on the same day. The former will on this occasion have the opportunity of inspecting an admirably equipped institution of the kind on thoroughly modern lines. There is much we can learn from our sisters across the sea, and we humbly trust that they may learn a little more of us on this occasion, as well as that they may enjoy their brief stay amongst us.

### Lectures Upon the Nursing of Mental Diseases.

By ROBERT JONES, M.D.Lond., B.S., F.R.C.S.Eng., M.R.C.P.Lond., Resident Physician and Superintendent of the London County Asylum, Claybury.

### LECTURE XII.

SLEEP is a physiological necessity of mental health, and the loss of it, in some cases of acute mental disease, is one of the most universally urgent symptoms. In some cases it is so extreme that the patient literally dies for the want of proper sleep. Sleep, normally obtained, is a periodic suspension of all conscious processes, and its depth, under healthy conditions, increases rapidly for the first hour, when it is deepest, then becomes gradually lighter until waking occurs. Dreaming is therefore physiological just before waking, when the brain begins to be active again. Sleep is necessary for exhausted nature, and the repair which is so urgent a necessity for the relief of fatigue and for efficient bodily restoration, is obtained during sleep. Repose is a greater regenerator than the change of scene so often advocated for cases of early or incipient insanity, and the tired faculties are better restored to healthy energy by tranquillity than by exertion. As to the physiological cause of sleep, all that can be safely stated is that it is due to organic conditions, probably to the using up of highly-organised protoplasm in the cells of the brain, which result from or accompany fatigue, prostration, or exhaustion of nervous matter. We know that fatigue predisposes to sleep, but we also know that when there is acute nervous prostration, as occurs in mental disease, marked wakefulness persists, possibly because a vicious circle is established, the worn-out brain-cells giving rise to fatigue products, which, accumulating in the blood, in turn react upon the already exhausted cells, exciting them to further action like beating a jaded horse. This probably corresponds to the irritability we so often experience when sleeplessness follows over-work and being "run down," and a stimulus applied to a sensitive nerve is much more felt at that period, when there is also less inhibition or self-control, permitting a lesser stimulant to fire it or to excite it into action. The peculiar haggard look of insomnia and the length of time the patient has been without sleep, will help the medical officer in whose care the case may be, to decide whether the treatment prescribed shall be that of *forced recumbence*, or *forced exercise*, and the assistance of the nurse—who will have to keep a "sleeping chart," to record the actual hours of sleep and the nature of the case—will greatly influence this decision. As a rule cases which are treated by the method of *forced recumbence*, are all those of acute exhaustion from mental diseases, and such cases as those of acquired neurasthenic insanity resulting from overstrain of mind and body, those who are senile and those suffering from the excitement accompanying the puerperal period. In cases of general exhaustion the bed treatment—with sleep, repose, and judicious feeding—is preferred to active exertion; for exercise that would be irksome, if compulsory, is not the best calmative to an exhausted and irritable nervous system. Even for sane persons who are "run down," present-day physicians urge a modified "rest cure," and nurses are often sought from among those who have received asylum training to carry out this treatment. Rest, feeding, and massage are the prime measures resorted to for procuring sleep. As a rule, melancholy cases and those of acquired hereditary insanity are best treated with forced exercise. This treatment is also a hygienic agency peculiarly suitable for some cases of mania, as the muscular activity affords a natural channel of discharge for pent-up nervous irritability which is so characteristically associated with the excitement of mania.

Loss of sleep is not only an effect of insanity, as may be seen in the rapid flow of ideas by night and day which occurs in cases of mania, and in cases of melancholia with much distress and agony of mind; but it is also a culminating and not infrequent cause of actual insanity in those of nervous temperament. The nurse, who has to keep a written record of the pulse, temperature, excretions, food consumed, medicine taken, and hours of exercise and sleep, must see that no bad habits are contracted from the remedies for sleeplessness, such as arise after morphia, opium, or alcohol used as hypnotics and soporifics. She must in this respect accurately and faithfully carry out the doctor's orders. Loss of sleep, when not actually causing insanity, has a serious effect upon the proper performance of the mental functions, the mind becomes sluggish, the senses are less acute, and reflex movements are more easily excited, as may be seen in the sudden jerks made by persons involuntarily—owing to the pressure of the bedclothes or to an uncomfortable position of the limbs—in the early stages of sleep. Although we are unable to state definitely to what sleep is due anatomically and physiologically, it is surmised that the cause is to be sought in some altered condition of the blood supply to the brain. We know that *active* tissues require *active* nourishing, and that in the brain there must be a vigorous reaction through the cerebral blood-vessels in response to any active call. Some have therefore described the state of the cerebral blood-vessels during sleep as a venous congestion, that, as in tumours of the brain there is pressure from interference with the normal circulation, and in consequence stupor, so in sleep there is also a venous engorgement, favoured by the slower respiration and the reduced action of all the functions which is known to occur in sleep. Others have stated that, physiologically, sleep is accompanied by anæmia, that observation through trephine holes in the skull has verified this, and that delicate instruments confirm this by recording a lowered blood pressure in the arteries of the neck which supply the brain with blood. There are, moreover, observers who affirm that certain conditions of the nerve cells and their very delicate fibre-processes have to do with sleep, that sleep is a nervous impression through these nerve cells, and two opposite rival theories have been advocated to explain this, the one considering sleep to be due to retraction of the small processes of nerve-cells which end in ramifications round other cells, such reaction leading to a partial separation of the fine terminations from the cells they surround, leaving a space which, so to speak, cuts off nerve-currents and so induces sleep (Lepine). The other view considers sleep to be concomitant with a greater projection outwards of all the fine nerve terminations round the cells which then touch each other more closely and intimately and so diffuse nerve impulses which waste their effect in a general loss; the state of waking, on the other hand, being considered due to retraction of the processes and gemmules, so that the nerve-wave is intensified by having to be concentrated through the few paths which remain open (Lugaro). These views are at the best only plausible theories and they cannot be accepted as in any way proved. Indeed, it is not even known that the changes described actually occur, or, if they do occur, that they are concomitants of altered function in the brain-cells, or that they are the causes of any functional conditions at all.

It is well known that during sleep certain mental disturbances are liable to occur, against which the nurse must be on her guard. Of these epileptic fits are the most important. The patient is often unconscious of the fits and will, in consequence, deny their occurrence, because the memory sleeps soundly, as the vague recollection of our own dreams often proves. It is only when certain symptoms remain, such as the biting of the tongue, wetting the bed —the urine during the fit being voided involuntarily—or a feeling of muscular soreness from tonic spasms, a headache, or a general feeling of *malaise* that the patient is reminded of the fit. The night terrors of children are, in all probability, forms of epilepsy occurring during sleep. It also happens that hallucinations giving rise to delusions or transitory mania may occur during sleep. The condition known as somnambulism or ordinary sleep-walking is known to most nurses, and such a state may itself give rise to insomnia. As sleep is so dependent upon the attention and services of the nurse, it has been entered into somewhat fully.

In mental diseases the nurse's duties in regard to sleep are to promote it by seeing that the day is properly spent and that the patient has a daily discipline in regard to food, occupation (if possible), exercise and diversion. As to food, the patient's meals should be regular, ample, and nicely served. The meal must not be hurried—a prolific cause of dyspepsia and sleeplessness, for sound sleep implies the perfect harmony of all the organic sensations. The nurse should encourage each patient to sit decorously at table, and to say grace before and after meals. Apart from any religious view, this act punctuates a meal, and this makes of it a ceremony or rite which implies order, method, and discipline. A short rest should follow a meal, then some occupation varied by diversion and exercise, for well regulated muscular exercise is one of the best hypnotics; neither work nor diversion should be too continuous or prolonged, as there may be a mental strain involved in both, and the change from the one to the other should be complete and thorough. In many melancholy patients a mere walk does not cause them to forget their imaginary misery. An effort should be made to engage them in some work with others. The "Colonial" feeling of working with others is the best diversion, and the day should be spent in a methodical and regular discipline, avoiding excitement and mental over-strain, for sleep itself is a regular function. Although in mental diseases loss of sleep may be due to worry, anxiety, and painful emotions, yet it may also be directly due to physical causes, such as dyspepsia, already referred to, visceral disorders, skin abnormalities, heat or cold, noise or too much light, and it is the duty of the nurse to modify these so far as possible. A quiet bedroom in a retired part of the house should be selected, there should be thorough ventilation of the room, as well as of the bedclothes. The bed should be well made, without crumpled sheets, inequalities, or lumps in the bedding, and the mattress should be easy to lie upon, and if possible should be wire-woven. No light should be allowed into the room, and no glaring fire. The temperature of greatest comfort is 60°, although when sleep is obtained, it is stated that the lower the temperature — even to freezing-point — the sounder the sleep. The night nurses in institutions receive instructions to wear soft felt boots or slippers at night—for quietude at night, desirable as it is for mentally healthy persons, is absolutely indispensable in the treatment of mental diseases; to open and close the doors quietly and to avoid flaring her lamp in the faces of the patients who are sleeping. The same precautions should be observed by the private nurse. Cold is often the cause of sleeplessness, and when the nurse is on her rounds a glass of hot milk or a cup of hot beef-tea with a crust of bread or a biscuit will often help the patient to procure sleep, and hot drinks are often a reliable antidote to the insomnia of general mental unrest. In senile cases, with sleeplessness and restlessness, alcohol is often administered to assist sleep, and the nurse must see that only the amount prescribed is given, and that the patient is not indulged in what might excite a dangerous craving and create a habit too difficult to break. Warm baths are occasionally prescribed for mental cases to induce sleep, and the nurse must observe all the usual precautions in bathing, viz., as to the heat of the bath, time of immersion, and care in drying. The amount of sleep varies with temperament and age, some require much less than others, but however short the duration the main thing to observe is, that all the precautions for sleep should have their regular methodical and punctual placing in the discipline of the day, and that sleep itself is a normal function of clockwork precision and regularity.

### Nursing in a Boys' School.

BY A CORRESPONDENT.

It was my first experience of nursing in a boys' school. I wondered if it would be a success. I was very kindly received by the head-master, who informed me that an "epidemic" of measles was threatening, the sanatorium being already full, with the exception of a few beds. I was to have charge of the west dormitory, where any fresh cases were to be sent. There were already 10 boys awaiting my arrival.

I was then shown to my room, really the "sewing-room," just inside the "west dormitory." Everything seemed to have been very carefully arranged, and appeared as comfortable as possible. We were well isolated from the rest of the school, and everything was convenient—a great point.

### My Numerous Patients.

As soon as I was ready after my journey, the matron came to introduce me to my patients. They were naturally rather shy just at first. The rash was already well out on four of them, who said they were feeling "jolly bad." The day after my arrival was Sunday, but the measles seem to require no rest, for by Sunday evening I had six new patients, and by the end of the week the measles had been so busy that my patients had increased to 30. Even had I felt inclined, it was no use complaining. Another nurse was out of the question, they were in such demand at that time. As may easily be imagined, I was hard at work the whole day and the best part of the night. The matron relieved me as much as she possibly could, and always managed to let me go out for at least an hour each day. The room was kept clean by a maid, and the under-matron generally helped me to make the beds, which was no easy task while the boys were unable to get out. I had to begin taking the temperatures very early, or I should never have got round before breakfast. After breakfast it took nearly the whole morning to wash all the boys, then there were medicines and nourishment to give, besides the other numerous things which always occur in sickness. However I managed to get along very well—boys make very good patients—and by this time we were the best of friends. When I went round the last thing to "tuck them in," the younger ones—they were all ages varying from 18 to six—would invite me to sit on their bed and tell them a story, and it was very gratifying to hear the big boys say "Thank you, Nursie, it's almost as good as being at home, you're a regular brick," which, I understand, is the very highest praise a boy can give one.

### Successful Cases.

I was very fortunate, only one of the 30 had a relapse, and he was a delicate little fellow at the best of times. He developed pneumonia and had to be removed to the sanatorium where he could receive more careful attention than I, with my other duties, had time to bestow upon him. However, he eventually recovered, and I had the pleasure of seeing him sitting up in bed the day I came away. When the boys were convalescent I really had a very pleasant time. We had the use of a large room which was beneath the west dormitory where those who were nearly well might spend the afternoon, it made a change for them and left the dormitory quiet for those who had not quite recovered. I used to pay the boys downstairs an occasional visit, to see they were not in mischief; but as a general rule I found that if a boy made a promise he very rarely broke it.

### Sunday Amusements.

Sunday was the most trying day during convalescence; still we managed to get along very well. One of the older boys had the privilege of being a "chapel reader," *i.e.* one who reads the lessons in turn in the school chapel. When the boys were quite ready I asked them all to sit down quietly, and then asked P—— to read the Lessons and Psalms, which he did very well. In the afternoon we had a chapter read by P——, and another boy asked us questions on the subject. It was astonishing how good the boys were and how interested they became, trying who could give the most correct answers. Ordinary days were no trouble, I asked the head-master if we might have some cards and games and one or two soft balls, which he readily agreed to. With the help of the cards and games we managed to have great fun. Nothing pleased the boys more than to get me to "play ball" or take a hand at "whist" or "nap." For counters we used haricot beans. They were also very fond of suggesting a game of "old maid" which was hardly fair to me, still it caused them tremendous fun, but I am rather afraid they were not quite straightforward over this game, for I was invariably the "old maid" much to their amusement.

### Practical Jokes.

The boys being schoolboys, of course went in for practical jokes. One very wet day they had quite decided that I should not go out for a walk, and had arranged their games for me to take part in them, so though I asserted my intention of getting "a blow," they tried their best to keep me in, and succeeded. When I went to prepare for my walk my bonnet was nowhere to be seen; I felt certain I had laid it on my bed. After much hunting about, I asked the boys if they had put it away anywhere, and they all denied having touched it, the real culprit—whom I did not miss from such a number—being in hiding under a bed to save being asked. I returned to my room for a final search, but it was still nowhere to be seen. I then went again to the dormitory and tried to assume a very stern manner, and told the boys that they must say at once what they had done with it. The culprit then appeared and asked me to promise that I would not go out if he told me where he had put my bonnet, otherwise, if I did not promise he would not tell. It was very wet and they evidently did not want me to go out, so I promised. He then said slyly, "Did you look under the chest of drawers?" Of course I had not; anyhow, there I found it, covered with dust, but otherwise uninjured.

### The Biter Bit.

Even jokes can be carried a little too far sometimes. One day a boy wanted to "pay off" another, and he thought the best way to do this would be to prepare what he termed "a deadly concoction"; he did not intend to harm the boy, but just wanted to make him feel sick. He waited my departure to tea, then took himself off to the cupboard where the medicine was kept—this, of course, should have been locked—but it was only a boot cupboard in the ordinary way. Then he prepared his "deadly concoction" as follows:—Brandy, raspberry vinegar, port wine, fever mixture, sal-volatile (fortunately only small quantities of these), milk, linseed-tea, and barley-water, filled up with orange-juice, making in all about a tumblerfull. Just as the dose was ready for administration I returned from tea. It was then a case of the "biter bit," for, to save discovery, the culprit drank the whole dose off himself! I should probably have never heard anything at all about this little affair if it had not made the boy feel very sick. He came to me looking really ill and asked if he might go to bed. I took his temperature, which was considerably above normal, and at once gave my consent. When he was comfortably settled in bed I asked him if he could in any way account for his sudden sickness. He would not tell me at first, but, after considering for some time, he said, "Oh, well, if you won't split I'll tell you. When he had told me the whole story, I naturally administered another dose to work off the ill effects of the first. This hardly met with the young gentleman's approbation. However, it had the desired effect, and by the morning he was quite himself again.

### Saying "Good-Bye."

I was only too sorry when the day of my departure arrived. I had spent such a happy time and it was altogether such a new experience for me, besides being quite a success. In spite of a football match which was taking place when I came away about a dozen of my late patients came to see me off. I am sure my fellow-passengers did not long wonder what my last case had been. The boys were most uproarious, and the last words I caught as the train puffed out of the station were, "Good-bye, Nursie; don't forget the measley chaps!" which was quite an unnecessary injunction.

### Croydon Guardians and Sick-room Cookery.

At a meeting of the Croydon Guardians on Tuesday, it was reported that an examination in sick-room cookery was held on May 13th. The report from the Examiner stated that it was exceedingly satisfactory, the following being the number of marks gained out of a maximum of 100:—Nurse Wright, 97; Nurse Morland, 94; Nurse Daintree, 93; Nurse Pickford, 90; Nurse Usher, 90; Nurse Knighton, 90; Nurse Bayes, 89; Nurse Bartholomew, 89; Nurse A. Williams, 87; Nurse Hart, 85; Nurse Jenner-Parsons, 83 Nurse Edwards, 81; Nurse Poole, 80.

The Examiner expressed herself, after the practical examination, as being particularly pleased with the work done, and the good method of working. The nurses had benefited so much by this course that she recommended that the classes be continued next winter, to other nurses. Dr. Wilson, medical superintendent, recommended that a special certificate be given to those nurses who had passed the cookery examination. The Infirmary Committee recommended that the classes be continued next winter for the benefit of other nurses, and that a certificate be granted to the foregoing nurses who had passed, in a form to be recommended by the matron. The board agreed to these proposals.

### Berlin Sisters at Work.

BY A CORRESPONDENT.

At a time when a number of English and American nurses are about to visit Berlin, a few details of one of the most interesting institutions in the German capital may be acceptable. The Bethany Deaconesses' Home, which, besides maintaining and staffing a hospital of 300 beds, sends forth 253 sisters to work in 49 "out-posts" or hospitals affiliated with the Mother Home, has always enjoyed Royal patronage and favour. It was founded in 1845 by King Frederick William IV. of Prussia, and the opening took place in 1847, the King being present on the occasion. He himself selected the name of "Bethany." In a very short time the number of patients amounted to 100, at which figure it remained for a little while. Changes in the administration of the municipal affairs of Berlin, which prevented the King from having free control of its exchequer, brought the Home into sore financial straits, from which it was only rescued by the intervention of his Majesty, who, besides confirming its title to be a "free charitable foundation of the Evangelical Church," guaranteed it an annual contribution of £3,000.

In 1854 a mortuary was erected. In 1856 the sisterhood was divided for the first time into three orders—deaconesses, novices, and nurse-probationers. In the following year a farm, with accommodation for 16 to 18 cows, was built in one portion of the grounds. The Home itself stands in the centre of 45 acres, so that ample space is afforded for the addition of various buildings.

In 1859 the King granted the sum of £3,750 for the purpose of erecting a Home of Rest for the sisters at a watering-place on the Baltic called Heringsdorf. This house, where seven of the sisters can be accommodated, bears the name of "Königsgabe" (King's Gift). It is in a charming situation, and has already strengthened and invigorated many of the sisters. The deaconesses have not been found wanting in times of war. During the Schleswig-Holstein campaign 18 of their number served in the military hospitals. In the war with Austria 41 of the Bethany nurses were at work. Thirty-seven were sent into the field during the Franco-German war.

On the occasion of the 25th anniversary of the foundation of the institution a sum of money was collected to build a Home where aged deaconesses might pass the evening of life not far from the beloved establishment where all their long years of service had been spent. In 1894 the deaconesses' training school was opened. Here young girls from 15 to 17 who feel a desire to become deaconesses are trained, and at 18, if they are still of the same mind, they become nurse-probationers.

One of the principal rules of the Home is that each sister must wait upon herself. In fact, all that has to be done in a sick room must be done by the sister. For those who cannot or will not work there is no room at Bethany.

The last annual report shows that the work is still making steady progress. At the end of the year there were in the Home 271 deaconesses, 68 novices, and 14 nurse-probationers, or a total of 353, as against 265 deaconesses, 64 novices, 21 nurse-probationers (total 350) in the previous year. Comment is made on the diminishing number of nurse-probationers, which is stated to be less than it has been for many years; the same state of things prevails also in all the other deaconesses' Homes. The two Homes of Rest for the sisters were crowded during the whole of the summer, and the greater part of the sisters were thus enabled to enjoy a long or short holiday. There were 21 pupils in the training school. At the end of the year there were in the hospital 149 male and 151 female patients.

In addition to the Bethany Sisters' Home of Rest at Heringsdorf, there is also a Home of Rest for delicate children, and here, during 1903, 66 children and 15 adults in need of rest and change were received. The "rest cures" lasted from July 3rd to August 7th, and from August 10th to September 11th; the second period had to be curtailed on account of the weather. Most of the parents paid for the maintenance of their children at the Home, but in the case of 12 little ones only the travelling expenses were contributed, all the rest being free.

### Appointments.

[No charge is made for announcements under this head, and we are always glad to receive, and publish, appointments. The information, to insure accuracy, should be sent from the nurses themselves, and we cannot undertake to correct official announcements which may happen to be inaccurate. It is essential that in all cases the school of training should be given.]

Bucknall Hospital, Stoke-on-Trent.—Miss Frances Hall and Miss Sarah Anne Mountford have been appointed charge nurses. Miss Hall was trained at the Isolation Hospital, Willesden, where she has since been staff nurse and sister. Miss Mounsford was trained at St. Marylebone Infirmary, London, and she has since been first assistant nurse at the Northern Hospital, Winchmore Hill.

Coventry City Isolation Hospital.—Miss J. E. Cooke has been appointed sister. She was trained at the Chorlton Union Hospital, Manchester, and has since been charge nurse at Prescot Infirmary, and sister at the City of Glasgow Fever Hospital, Ruchill. She holds the L.O.S. certificate.

Hanley, Stoke, and Fenton Joint Hospital Board — Miss Amice Elizabeth Brewer has been appointed charge nurse. She was trained at Newport Infirmary, and has since been staff nurse at Chester Isolation Hospital. During 1903 she had charge of the Chester Small-pox Hospital.

Isolation Hospital, Salisbury.—Miss M. Gray has been appointed matron. She was trained at Guy's Hospital, London, and afterwards was attached to the private nursing institution at Guy's. She has since been charge nurse at the Salisbury Isolation Hospital.

Royal National Hospital for Consumption for Ireland. — Mrs. Violet Wyatt (*née* Müller) has been appointed lady superintendent. She was trained at the Western Infirmary, Glasgow. She has since been sister of out-patients' department at the Royal Hospital for Sick Children, Edinburgh, sister-in-charge of the Huntingdon County Hospital, matron of the District Hospital, Bushey Heath, Herts, and sister-in-charge of Pavilion I. at the Consumption Sanatorium of Scotland, Bridge of Weir.

St. Bartholomew's Hospital, Rochester.—Miss H. Noble has been appointed night superintendent. She was trained at the Royal Victoria Hospital, Belfast, and has since been sister of female surgical wards at St. Bartholomew's Hospital, Rochester.

Woodlands Convalescent Home, Rawdon, Leeds.—Miss Gertrude MacCarthy has been appointed matron. She was trained at Brownlow Hill Infirmary, Liverpool, and has since been charge nurse at the Park Hospital, Hither Green, S.E., senior sister at the Borough Sanatorium, St. Helens, and assistant-matron at the Convalescent Home, New Brighton.

### The Central Midwives Board and the Irish Training Schools.

A meeting of the Central Midwives Board was held on Thursday last week, Dr. F. H. Champneys in the chair.

The further consideration was resumed of letters from the Royal Academy of Medicine in Ireland; the Rotunda Hospital, Dublin; the Coombe Hospital, Dublin; and the Belfast Maternity Hospital, asking for a modification of the Board's rules, so as to facilitate the admission of Irish-trained pupil-midwives to the Board's examinations.

After discussion, it was resolved that the Privy Council be asked to sanction the appending of the following Note to Rule C.I. (2):—

*Note.*—A certificate to the effect that the candidate has nursed 20 lying-in women during the *eight* days following labour will be accepted in place of the above in cases (1) where the course of special training in a hospital has extended over a period of six months, or (2) where a course of three months' special training in a hospital has been preceeded by a full course of training in general nursing.

It was also resolved that the Privy Council be asked to sanction an alternative form of certificate under Form IV. in the Schedule, to meet the modification contemplated by the above Note. It was decided that reporters of the recognised medical and nursing journals be invited to attend the meetings of the Board.

Chester Benevolent Institution was approved as an institution for the training of midwives under Section C. of the rules.

The following were approved as teachers under Rule C.I. (3):—P. E. Barber, M.R.C.S.; Robert Boxall, M.D.; H. Spencer Browne, M.R.C.S.; H. Caudwell, L.R.C.P.; Francis Chown, M.B.; F. W. S. Culhane, M.R.C.S.; J. W. Fordham, sen., M.R.C.S.; G. R. Harcourt, M.B.; David Charles Rayner, F.R.C.S.; William Shaw, L.R.C.P.; and A. L. Hall Smith, M.R.C.S.

The following were approved as certified midwives for the purpose of signing Forms III. and IV. under Rule C.I. (2):—S. A. Messenger, Katherine Twining, M. A. Webster, and L. E. Young.

After consideration of applications for certificates the names of 878 women were passed under Section 2 of the Act, and ordered for entry on the roll. The total number of midwives now enrolled is 5,330.

### Everybody's Opinion.

[Correspondence on all subjects is invited, but we cannot in any way be responsible for the opinions expressed by our correspondents. No communication can be entertained if the name and address of the correspondent are not given as a guarantee of good faith, but not necessarily for publication. All correspondents should write on one side of the paper only.]

### STATE REGISTRATION.

"A Provincial Nurse" writes: Having read with interest Miss Lückes' paper in "The Nineteenth Century and After," against the Bills for the Registration of Nurses, as a nurse who is strongly in favour of the movement, I would like to ask those who have signed their names against reform, if they are perfectly satisfied with the present system, methods, and general efficiency of the nursing profession? If not, what do they suggest instead of registration? I cannot believe that people holding such responsible posts in the nursing world as many of these do, would sit down satisfied to oppose a method of reform unless they have some better scheme in view. It would be of great interest to have some definite answer from them as to their views in the matter and why they are so strongly opposing an attempt at reform without putting forward a better suggestion.

### REGISTRATION AND MALE NURSES.

Mr. George Bloomfield, a certificated male nurse, writes: May I be permitted space in your valuable paper to say a few words on the subject of registration for male nurses. In The Hospital recently the present attitude of male nurses is described as one of calm indifference, but this, I venture to say, is the only attitude possible under the present circumstances. At present male nursing is in a very unsatisfactory state, and the male nurse is looked down upon as being much inferior to his female colleague, but my experience is that men have proved themselves to be very capable nurses when they have had a proper training, and they are just as much a necessity as trained women. The unaccountable prejudice against us as a class is largely owing to the fact that many of these unskilled men who are engaged in nursing and who call themselves trained nurses have not had the opportunity to have an efficient training, and therefore cannot be trusted to undertake grave responsibility such as a nurse often has to take. If registration came into vogue the standard of training of many of these men would be found sadly wanting, and they would have to drop the word "nurse" and substitute that of "attendant." Under present circumstances State registration for male nurses would be of little use as there are so few hospital-trained male nurses in practice in this country. The remedy I propose is that the great general hospitals admit to their training schools male probationers after the manner of the "National Hospital for the Paralysed and Epileptic." There ought to be no difficulty for any large hospital to introduce male probationers, say in one of their wards which could be set apart for them with a suitable sister at its head and an extra man or two for emergency work in the other wards, such as catheters, or to "special" a violent patient, or help to lift an especially heavy patient. It would soon be found that male nurses are of incalculable value in hospitals, and they would soon repay any initial outlay which the hospital might make. These men should be subjected to the same rigid discipline as the female probationers, could attend the same lectures and work for the same certificate, and be answerable to the matron for their general good conduct. These conditions act smoothly at the National Hospital, and there are no practical difficulties caused by both sexes working together, and frequently, when necessary, male and female nurses work together in the same ward with perfect harmony. In conclusion, I beg to emphasise that when the great general hospitals open their wards to male nurse, then and not till then will State registration be of any avail.

### DR. BOXALL AND THE MIDWIFE'S AGE.

Dr. Boxall, 40 Portland Place, writes: The paragraph which you were kind enough to insert in The Hospital of May 21st, concerning the age of candidates for examination as midwives, does not accurately convey the remarks which I endeavoured to make clear to you by telephone. The minimum age of 21 is now laid down by law (see regulations of Central Midwives Board), and is the same as that which has been adopted by the Obstetrical Society of London for many years. It has not been found in practice disadvantageous. As to the maximum age of candidates, no general regulations exist, but as a matter of experience in training 40 has been found none too young as a rule; though at the General Lying-in Hospital the authorities have for the present, and as an experiment, recently sanctioned 45 as the upward limit. This has been done in order to afford an opportunity of training to as many as possible of those women who are willing to improve their position, instead of being content merely with inclusion of their names on the "roll" as *bonâ-fide* midwives.

[The original summary of Dr. Boxall's speech was too compressed to make it clear that he was referring to facts, not expressing opinions. Hence the misunderstanding. But we have always thought that 21 is too early for anyone to qualify as a midwife.—Ed. Hospital.]

### Echoes from the Outside World.

### Movements of Royalty.

On Thursday last week the King and Queen with Princess Victoria left Windsor during the morning to open the Royal Military Tournament at the Agricultural Hall. At Paddington they received an enthusiastic reception, drove to Buckingham Palace to lunch, and then proceeded in an open carriage drawn by four horses with postilions and outriders to Islington. The most important item on the programme was the historical pageant. Mail-clad trumpeters introduced artillerymen of the Crécy period, with a red cross upon their breast. They were accompanied by a "bombard," dated 1400, from the Rotunda Museum at Woolwich. These were followed by Tudor gunners, soldiers of Cromwell and of the Restoration period, "A Traine of Artillerie" of Marlborough's time, and a gun carriage which played a part in the siege of Gibraltar. Gradually the show worked its way to our own time, and the heroes of the Peninsular War, of Waterloo, and the Crimea as well as South Africa were greeted with applause. Representatives from our Indian Empire were not forgotten, and the mules, camels, and elephants increased the picturesqueness of the scene. Their Majesties appeared to derive much pleasure from the parade, which reached a climax when the Royal salute was given, and the whole audience rose at once to the strains of the National Anthem.

On Friday a large party, including the King and Queen, Princess Victoria, the Prince and Princess of Wales, and Prince Edward of Wales, went in three motor cars to pay a visit to the Kneller Hall Royal Military School of Music at Hounslow. The orchestra consisting of 160 students and pupils played on a raised platform. The répertoire included the overture to "Rienzi," a selection from "Aïda," Elgar's "Salut d'Amour," and a march composed by Student C. Landrock. The uniforms represented every regiment of the British Army, including the East African and West Indian Regiments.

On Tuesday morning the King and Queen visited the flower show of the Royal Horticultural Society in the Inner Temple Gardens. No single exhibit escaped the notice of their Majesties, but they were attracted to several collections by reason of their special beauty. The King singled out the old black tulip for praise, and the Queen some pink spikes of the Eremurus robustus, also a collection of dwarf Japanese trees and some very fine rhododendrons. Their Majesties remained some time in the tent where the orchids and roses were displayed.

### The Alake at Buckingham Palace.

On Sunday the Alake of Abeokuta attended service at Westminster Abbey, both morning and evening, and sat in the stalls near the Dean. On Monday morning he was received by the Colonial Secretary, and soon after 1 o'clock drove in an open carriage to Buckingham Palace. His great desire was to have an interview with the King. His Majesty, who was in field-marshal's uniform, received the Alake and his suite in the Throne Room. The visitors, advancing to the Royal presence between two rows of gentlemen-at-arms, made three separate pauses, kneeling at each, and then stood in front of the throne while the formal presentation was made, the native secretary acting as interpreter. The King's greeting was most cordial. He inquired after the Alake's health, and said that he was greatly pleased at the prosperity of his country and at what he had heard of him personally. The Alake, in reply, said that he greatly appreciated his Majesty's gracious reception, and that he was proud to look to the King as his Sovereign protector. The Alake wore on his head the State crown of gold lace with three lizards; he was attired in a robe of native workmanship, consisting of a long gown of ruby velvet almost entirely covered with gold embroidery; on his breast was a great silver star, and round his neck an enormous gold necklace.

### Japanese Victory at Kin-chau.

The news that the Japanese, after a battle lasting five days, last week defeated the Russians and captured Kin-chau, has been officially confirmed. The fighting was of the most desperate kind, and it is stated that the Russians left upwards of 500 dead on the field, the total of the Russian losses being estimated at about 2,000. The Japanese commander, who captured 68 guns and 10 machine guns, estimate the casualties on his own side at about 3,500. When the Japanese eventually forced their way into the lines the Russians retreated in confusion, burning their magazines at Ta-fang-shin. The Japanese have since occupied Liu-shu-tan, capturing in the occupation four guns and 56 waggons, and dispersed 2,000 Russian cavalry at Ai-yang-cheng. A Japanese detachment occupied Dalny on Monday. Over 100 warehouses and other buildings were found uninjured, and nearly 300 railway cars are still usable. All the docks and piers, except the great pier, are unharmed.

### The British Forces in Tibet.

From Gyangtse it is reported that last Thursday a column under Colonel Brander attacked and cleared a village in Pulla, which was held by about 300 Tibetans. Only about 50 of the enemy escaped, 40 surrendered, and the remainder were killed or wounded. Lieutenant Garstin was killed in the fight, three other officers were wounded, and of the rank and file three were killed and eight wounded.

### Conscription Recommended by a Royal Commission.

The report of the Royal Commission on the Militia and Volunteers, which was issued last Friday, is remarkable for the fact that the Duke of Norfolk and a majority of his colleagues, after recommending the adoption of certain measures to increase the efficiency of the Militia and the Volunteers, state that even if their recommendations were carried out, they cannot assert that these forces would be equal to the task of defeating a modern Continental army in the United Kingdom. They are convinced that only by training the whole able-bodied male population to arms, the training being given in a period of continuous service with the colours, not necessarily in barracks, by a body of specially educated and highly trained officers, can an army of home defence, adequate in strength and military effiiency to defeat an invader, be raised and maintained in the United Kingdom.

### The Children's Salon.

The fourteenth annual competition of the Children's Salon took place on Saturday, at Caxton Hall, Westminster. Owing to the fact that the proprietors of *The Gentlewoman* defray all the costs of printing, correspondence, office, etc., it is correctly described as a "charity without expenses." There are already cots in the Victoria Hospital for Children, the Cheyne Hospital, Chelsea, the North-Eastern Hospital, the North-West London Hospital, and the Children's Hospital in Great Ormond Street, endowed by the Salon Workers, and now "the children of the rich," by way of helping "the children of the poor," are in process of establishing a sixth cot at the East London Hospital for Children at Shadwell. The prizes were to have been distributed by Princess Henry of Battenberg, but unfortunately she was prevented from attending, and at short notice Princess Louise Augusta of Schleswig-Holstein took her place. The programme included a singing contest, recitations, and dancing.

### A Book and its Story.

### THE TOPIC OF THE DAY.*

Japan, being at this moment the country of most interest to the civilised world, an immediate demand has arisen for all books on the subject. Mr. Diósy is a reliable authority, and his lucid and exhaustive treatment of the subject in his popular book, "The New Far East," shows that his desire to impart a knowledge of the truth about the New Far East, in some of its principal aspects, especially those in which the nations of the West, and all English-speaking peoples, are most interested, has been happily realised. The book first appeared in 1898, and the stirring events which have occurred since then give an added interest to the contents. New editions followed quickly, the present one being the fourth. Three historical changes have come about since the issue of the second edition. We quote from the preface:—

1. "The alliance of Britain and Japan, whereby British policy in the Far East has, at last, entered on the 'clear course' which I have so long strenuously advocated, and which is treated of in Chapter X.

2. The war between Russia and Japan, the inevitable struggle for preponderance in the Far East, and Japan's immediate exposure of the hollowness of the Muscovite bluff.

3. The unmistakable symptoms of the gradual awakening of China under Japanese impulse, the Chinese turning in their extremity, more and more towards their old foes, whose just and humane conduct in the repression of the 'Boxer' outbreak of 1900 so deeply impressed the Celestial mind."

The author has employed the famous Tokio artist, Kubota Beisen, to illustrate "The New Far East." A reproduction of a cartoon, "The Yellow Peril," designed by H.M. the German Emperor, and a specially drawn map, showing the advance of the Russian frontier line since 1858, adds to the interest and value of the book, in which no detail has been disregarded which will assist the reader in realising the importance of the question which the author has made a life study. Many instances are given of the humanity of the Japanese to the wounded Chinese after the capture of Port Arthur in November 1894, and throughout the war, while that part of the book dealing with the organisation and equipment of the Japanese field hospital will have special interest for the readers of The Hospital. "The admirable arrangements for keeping the troops at the front in good health and for saving their lives and alleviating the sufferings of the sick and wounded, were due not only to the perfect organisation of the Army Medical Service, adapted from the German model, and of the Naval Medical Department, originally formed and trained under British inspiration, but also to the well-directed efforts of the Red Cross Society of Japan. This noble organisation, with a membership of over 200,000, with branches in every part of the Empire, and an annual income of over half a million yen, stands under the immediate and active patronage of the Imperial family, ever foremost in good works. During the war it expended nearly 4,000 yen on its merciful work (the average value of the silver yen was, in 1898, 2s.), sending three hospital parties, consisting of surgeons, dressers, apothecaries, matrons, trained nurses, accountants, clerks, porters, cooks, and even, grim detail, 'instrument sharpeners,' to the seat of war, each party being fully equipped with every requisite for the treatment of 200 patients." The Japanese Hospitals were often crowded with the wounded Chinese who received the same skilful treatment and gentle nursing as the Japanese soldiers. The Chinese had no hospitals, and by them a wounded comrade was treated as an encumbrance, to be stripped of his arms, accoutrements, and uniform, and left to die where he fell. The contemptuous term of "Dwarf" was used by the Emperor of China in his proclamations during the war, when referring to the Japanese. In what strong contrast the humanity of the "Dwarfs" appeared against the indifference to the sufferings of their soldiers, shown by the Chinese, the following passage will illustrate: "The wounded Chinese were picked up from the blood-stained snow, borne carefully to their dressing stations, and then to the clean, airy field hospital, where calm, skilful 'dwarf' surgeons operated, and then on to their great roomy, tidy, base hospital, where gentle 'dwarf' nurses tended them with tiny soft hands. Well might they think, as many Chinese did, that the Japanese bullet had killed them outright, for was not this Paradise? Surely, those gentle women with the low sweet voices and kind eyes, must be the angels who sprinkle the lotus with nectar in the Buddhist heaven. This must be the commencement of a new and happier existence—plenty of food, nothing to do, clean clothes, and a bright airy palace to live in!" Thus the convalescent Chinese pondered, and he was, as a rule, quickly convalescent, for the wounds of the Far Eastern people heal with astonishing rapidity, owing to their living chiefly on vegetarian diet, or, at all events, eating but little meat, and to their drinking as a rule only a moderate quantity of intoxicating liquor, and that not of a very injurious nature. If the wounded Chinese was not absolutely beyond human help his cure was almost a matter of certainty, for of all the marvels Japan exhibited to an astonished world during the war the greatest was her medical service afloat and ashore. "Not a life was lost on the Japanese side that medical service would have saved," said Surgeon-Major-General Taylor, of the Royal Medical Corps, in a lecture delivered at Aldershot shortly after his return from the seat of war. Mr. Diósy has much to say on the subject of the social condition of Japanese women, on education, new Japan, and the new woman. "The new woman of Japan represents a class that is growing, very slowly, it is true, but still growing, of women who are determined to treat men on an equal footing. It is quite certain that they will meet with resolute opposition. It is not that the men of New Japan have any rooted objection to the intellectual development of their womankind. Every effort in that direction enjoys the patronage of the ladies of the Imperial family, especially the Empress . . . but it is when we begin to inquire into the reasons for this enthusiasm for female education that we perceive the vast difference between the Japanese point of view and our own." The majority of Japanese men desire to see their women well educated because "the educated woman can better perform her duties as a daughter-in-law—the first consideration—as a wife, as a mother, and a daughter. The Japanese women, in spite of long subjection to the will of mothers-in-law and husbands, retain, under a gentle exterior, an iron will, and the meek little ladies, on occasion, become heroines who tower head and shoulders above the ordinary run of womankind . . . in hospitals, and at home, giving proofs of fearless devotion, stoical resignation, and of ardent patriotism." Mr. Diósy acknowledges his indebtedness to Miss Mabel Bacon for that portion of his book which treats of Japanese girls and women.

* "The New Far East." By Arthur Diósy. Cassell and Co. 3s. 6d.

### Notes and Queries.

**REGULATIONS.**

**The Editor is always willing to answer in this column, without any fee, all reasonable questions, as soon as possible.**

**But the following rules must be carefully observed:—**

**1. Every communication must be accompanied by the name and address of the writer.**

**2. The question must always bear upon nursing, directly or indirectly.**

**If an answer is required by letter a fee of half-a-crown must be enclosed with the note containing the inquiry.**

*Nursing in the Riviera.*

(69) Kindly let me know to whom I should apply for particulars of the institute in London which sends out nurses to the Riviera from May to October.—*A. M. M.*

Write for particulars to the Lady Superintendent of the Nice Nursing Institute—formerly the Hollond Institute in London—Villa Pilatte, Avenue Desambrois, Nice.

*Agency.*

(70) Can you recommend an agency through which one might apply *re* partnership or sale?—*Mrs. B.*

We do not give private recommendations.

*Home for Babies.*

(71) Will you kindly tell me of a home or institution where babies are taken who are not orphans?—*C. A.*

We do not know of any institutions which undertake to relieve parents of their responsibility.

*St. John Ambulance.*

(72) In making application to be received as probationer, will mentioning the fact that I hold the St. John Ambulance Medallion be any recommendation?—*Ethel.*

We are afraid not.

*Housekeeping.*

(73) Is there any hospital where I could obtain three months' training in housekeeping either by giving my services, or by paying a fee?—*Sister.*

Possibly you may hear of one through an advertisement.

*Hospital Training.*

(74) Will you tell me if there are good recognised hospitals in London which give a two years' certificate? Is a nurse holding a two years' certificate eligible to take any good posts after leaving her training school?—*Birdie.*

See "The Nursing Profession: How and Where to Train," for lists of training schools and terms. A two-year certificate is not a sufficient qualification for the best posts.

*Specialist.*

(75) I should be glad if you will give me the name and address of a medical man who has made a speciality of cases relating to sex.—*W. R.*

We never give private names and addresses.

*Training Probationers.*

(76) What is the best book to help one in training probationers?—*L. C. B.*

"Hospital Sisters and their Duties," by the Matron of the London Hospital (price 2s. 6d.), is highly spoken of; whilst "Nursing, Its Theory and Practice," by Percy G. Lewis, M.D., M.R.C.S., L.S.A. (price 3s. 6d.), and "Nursing," by Isabel Adams Hampton (price 7s. 10d. post free), are old-established favourites. Either of these can be obtained from The Scientific Press.

*Directories of Nursing Homes.*

(77) I hear that there are agencies where nursing homes are recommended; can you very kindly supply me with any addresses?—*M. W. W.*

Will you please inform me if G—— H——, etc., is a "registered" home for nurses; and also state if there is a directory where I can get such information as regards that and other homes?—*C. G. E.*

There is no "directory" of nursing homes; neither are such homes and agencies "registered."

*Uncertificated.*

(78) I have had three years' training in a large London infirmary, but, failing to pass my examination, I have no certificate. I have also had 18 months' experience in fever nursing. Will you kindly tell me how I could secure a post in the nursing world? Should I train again, and if so, where could I train?—*G. J.*

Would not the matron under whom you were trained advise you? Some nursing homes would receive you. If you decide not to train again you had better advertise.

*Midwifery Certificate. Attending to the Dead.*

(79) If a general nurse studied midwifery, attending cases with a doctor, could she obtain certificate without further training? 2. Is it not the duty of the nurse, and not of the wardmaid, to comb heads, wash the dead, etc., even though there be but one nurse on duty in the infirmary?—*A Nurse.*

Write full particulars and ask the advice of the Secretary of the Association for Promoting the Training and Supply of Midwives, 12 Buckingham Street, Strand, W.C. 2. These duties certainly are frequently discharged by nurses, but if the nurse has more important work to do, they might safely be assigned to a suitable assistant.

*Notice.*

(80) Is a nurse, who has been engaged verbally for 12 months at a definite salary, bound to take a month's notice four months before the expiration of her engagement?—*Ecos.*

Yes; if she is paid monthly, unless she has a written agreement to the contrary.

*Hospitals v. Infirmaries.*

(81) Are infirmary-trained nurses (three years' certificate) equal in every respect to hospital-trained nurses?—*E. E. P.*

It depends entirely upon the hospital and the infirmary.

*Sanitary Inspector.*

(82) Can you tell me how to obtain information about the post of lady sanitary inspector?—*N. R.*

Will you kindly tell me what lady sanitary inspectors are supposed to know, and at what age she can enter the profession?—*F. J.*

Apply to the Secretary of the Sanitary Institute, Margaret Street. W., for all particulars.

*Invention.*

(83) I and a friend have hit upon a new device for a bed-rest, obviating many of the defects of the present patterns; which firm would be most likely to undertake the manufacture and sale of the same?—*Fourth of July.*

We cannot say. First get a provisional patent for your invention, and then submit drawings, etc., to the principal makers of invalid appliances. This you can ascertain by studying the advertising columns.

*Paris.*

(84) I should be glad if you will give me the addresses of one or two nursing institutions in Paris where a nurse with between two and three years' hospital experience, but uncertificated, might apply for work.

There are no institutes for supplying English nurses in Paris. You might apply to the Hertford Hospital.

*School Nurse.*

(85) I am very anxious to obtain a post as nurse in a boys' school or "house." I should be very glad if you will kindly tell me to whom to apply for particulars, and how I could find out where there is a vacancy.—*Nurse K. S.* and *K. M. L.*

Make your wants known by, and study, advertisements.

*Registered Midwife.*

(86) I wish to be registered as a midwife; will you kindly tell me what steps to take?—*Pandora.*

Write to the Secretary the Central Midwives Board, 6 Suffolk Street, Pall Mall, S.W.

*Special Certificate.*

(87) I have a three years' certificate from a women's hospital, and have also been sister there; but I find that I cannot obtain another post as sister without a three years' general certificate. Would it be of any advantage to train for one year as paying probationer in a large London Hospital? Would that certificate, along with my own, be of any value to me in the future?—*Alice.*

The matron at any of the large London hospitals at which you applied for training would be willing to advise you as to the best step to be taken in your present difficulty.

**Important Nursing Textbooks.**

"Nursing Profession: How and Where to Train." 2s. net; 2s. 4d. post free.

"Nurses' Dictionary of Medical Terms and Nursing Treatment." By Honnor Morten. 2s. post free.

"Nursing." By Isabel Hampton. 7s. 6d. net; 7s. 10d. post free.

"Surgical Ward-Work and Nursing." By Dr. Alex. Miles. 3s. 6d. net; 3s. 10d. post free.

"Outlines of Insanity." By F. H. Walmsley, M.D. Popular Edition. 1s. 6d. post free.

"Notes on Pharmacy and Dispensing for Nurses." By C. J. S. Thomson. 1s. post free.

The above works are obtainable of all booksellers, or direct from The Scientific Press, Limited, 28 & 29 Southampton Street, Strand, London, W.C.

### A Bag of Hay.

BY A MATRON.

[The following experience is written because it is so often difficult for young nurses to be considerate and tender as to the whims and fancies of the sick. It is a proof of how the granting of a whim—which could do no harm—resulted, under God, in the saving of a patient's life:—]

He was an old farmer, and had been suffering from a bad attack of enteric fever, and only at the end did his relations consent to have him moved into the hospital, which was some twelve miles distant from his home. They brought him, lying on a mattress in a cart, and when he arrived the poor man was well-nigh exhausted, whilst the wife and the sons were weeping. We quickly got him into bed, not daring in his exhausted condition to do any of the needful washing. The next day it took us several hours to take off even the upper layer of weeks of perspiration and powder, for soap and water had never touched him. Gradually, however, and bit by bit only, we got him into the state of regulation cleanliness! He had empyema we found, as well as huge bed sores, and he was very delirious. It was a battle to get him to take sufficient nourishment, sleep seemed to have forsaken him, his strength was almost gone, and the doctor looked grave and shook his head. The poor wife was allowed to remain with him, helpless in her sorrow. He was a tall man, and nothing would induce him to lie straight in his bed. He insisted on putting his head on the locker and his feet out at the opposite corner. "Let him lie as he likes," the matron said, "never mind the look of the ward." One day he opened his eyes, and looked around. "What is it?" we asked. "A bundle o' hay, if ye'd only gimme a bag o' hay for my head I'd sleep." Quickly the matron sent some one out for a "bag o' hay," and when it came we took it up to him. "Here's your hay, Mr. ——. Now where will you have it?" "Oh, God bless ye for it, put it under my head." So on to the locker the "bag o' hay" went, and he nestled his head affectionately down on it, while the gaunt feet and ankles as usual were out at the opposite corner on a chair with a pillow on it, with blankets to cover him. And he slept, a long good refreshing sleep, and woke up after some hours, during which he had been given milk from his "taypot" as he called the feeding cup, without being roused.

"'Twas the bundle o' hay," he said; "I knew I'd sleep if I could get my head on the hay." And from that day he steadily improved, though he would never sleep again on anything but his "bag o' hay." His next craze was for air and sunshine. It was useless to argue that the windows were open, and air coming in, and that he must wait a little longer to be carried out into the large garden which surrounded the hospital. "If ye don't put me in the air of the hivens, how can I get well? Look at my hands, they're like a lady's, I nivver had such hands before." And he was ready to cry at the "white lady's hands." So we begged permission from the doctor, and carried him down on the stretcher and laid him in the sunshine, "in the air of the hivens," and carried him back, long before he considered it time. After a day or two he insisted on having his "pants," and our "spoilt baby," as we called him, was allowed to be "dacently dressed," and to lie on a sofa in the garden and try to brown his hands in the sun. He was discharged "cured," a few weeks later, looking forward to being able to brown his hands in real earnest again before long.

"'Twas the bag o' hay," he said; "thanks be to God and ye." And the poor wife knelt in gratitude to call down the blessings of Hiven. "An' may your bed be in Hiven, and one to spare for a friend in need," was her parting blessing as triumphantly they drove away.

### For Reading to the Sick.

"I AM WITH YOU."

We cannot see before us,
But our all-seeing Friend
Is always watching o'er us,
And knows the very end.

What though we seem to stumble,
He will not let us fall;
And learning to be humble
Is not lost time at all.

What though we fondly reckoned—
A smoother way to go
Than where his hand has beckoned,
It will be better so.

What only seemed a barrier,
A stepping stone shall be;
Our God is no long tarryer,
A present help is He.

And when, amid our blindness,
His disappointments fall,
We trust his loving-kindness,
Whose wisdom sends them all.

*F. R. Havergal.*

At the Last Supper our Lord said: I am the Vine, ye are the branches," choosing this as the last picture of Himself, the last expression of His nearness to us, and of our dependence on Him. Let us gather some thoughts from this picture. First of all the wood of the vine is the plainest and least attractive of all woods. It is very crooked and unpleasant to the touch, and only useful for bearing grapes Our Heavenly Father is the husbandman and He works, He prunes, He directs the young branches and tendrils. He does it all in love for their good, but His operations are often painful, yet only by means of them can the vine continue healthy and produce its fruit, and fresh leaves shoot forth, giving promise of fruit. These leaves are patience, meekness, self-control, and humility, which come to protect the fruit, to adorn the vine and set forth its fruit in splendid array. And, lastly, the fruit grows slowly and ripens and is ready for the Master. Then it must first be trodden down in the wine-press. None but He can say, "I have trodden the wine-press alone:" but we must tread it too, though hand in hand with our truest Friend "through many tribulations.

"He has taken your soul," says a spiritual writer, "as a wild vine and set it in the richest soil and sunniest corner of His vineyard, and watered it with the dews of actual graces from above and from below with the streams of living water beside which it is planted: He has digged round the roots by sorrows and humiliations designed to soften and loosen the affections; and has pruned and trained the branches over the walls of His house, that He may find fruit in due season and in full measure, and may glory and rejoice in the tree which by its fertility does credit to Him, that He may not be disgraced by it and say: 'Cut it down, why cumbereth it the ground?'"—*Anon.*

